# CTCF *cis*-Regulates Trinucleotide Repeat Instability in an Epigenetic Manner: A Novel Basis for Mutational Hot Spot Determination

**DOI:** 10.1371/journal.pgen.1000257

**Published:** 2008-11-14

**Authors:** Randell T. Libby, Katharine A. Hagerman, Victor V. Pineda, Rachel Lau, Diane H. Cho, Sandy L. Baccam, Michelle M. Axford, John D. Cleary, James M. Moore, Bryce L. Sopher, Stephen J. Tapscott, Galina N. Filippova, Christopher E. Pearson, Albert R. La Spada

**Affiliations:** 1 Department of Laboratory Medicine, University of Washington Medical Center, Seattle, Washington, United States of America; 2 Program of Genetics and Genome Biology, The Hospital for Sick Children, Toronto, Ontario, Canada; 3 Department of Molecular Genetics, University of Toronto, Toronto, Ontario, Canada; 4 Fred Hutchinson Cancer Research Center, Seattle, Washington, United States of America; 5 Department of Neurology (Neurogenetics), University of Washington Medical Center, Seattle, Washington, United States of America; 6 Department of Medicine (Medical Genetics), University of Washington Medical Center, Seattle, Washington, United States of America; 7 Center for Neurogenetics & Neurotherapeutics, University of Washington Medical Center, Seattle, Washington, United States of America; Medical Research Council Human Genetics Unit, United Kingdom

## Abstract

At least 25 inherited disorders in humans result from microsatellite repeat expansion. Dramatic variation in repeat instability occurs at different disease loci and between different tissues; however, *cis*-elements and *trans*-factors regulating the instability process remain undefined. Genomic fragments from the human spinocerebellar ataxia type 7 (SCA7) locus, containing a highly unstable CAG tract, were previously introduced into mice to localize *cis*-acting “instability elements,” and revealed that genomic context is required for repeat instability. The critical instability-inducing region contained binding sites for CTCF—a regulatory factor implicated in genomic imprinting, chromatin remodeling, and DNA conformation change. To evaluate the role of CTCF in repeat instability, we derived transgenic mice carrying SCA7 genomic fragments with CTCF binding-site mutations. We found that CTCF binding-site mutation promotes triplet repeat instability both in the germ line and in somatic tissues, and that CpG methylation of CTCF binding sites can further destabilize triplet repeat expansions. As CTCF binding sites are associated with a number of highly unstable repeat loci, our findings suggest a novel basis for demarcation and regulation of mutational hot spots and implicate CTCF in the modulation of genetic repeat instability.

## Author Summary

The human genome contains many repetitive sequences. In 1991, we discovered that excessive lengthening of a three-nucleotide (trinucleotide) repeat sequence could cause a human genetic disease. We now know that this unique type of genetic mutation, known as a “repeat expansion,” occurs in at least 25 different diseases, including inherited neurological disorders such as the fragile X syndrome of mental retardation, myotonic muscular dystrophy, and Huntington's disease. An interesting feature of repeat expansion mutations is that they are genetically unstable, meaning that the repeat expansion changes in length when transmitted from parent to offspring. Thus, expanded repeats violate one major tenet of genetics—i.e., that any given sequence has a low likelihood for mutation. For expanded repeats, the likelihood of further mutation approaches 100%. Understanding why expanded repeats are so mutable has been a challenging problem for genetics research. In this study, we implicate the CTCF protein in the repeat expansion process by showing that mutation of a CTCF binding site, next to an expanded repeat sequence, increases genetic instability in mice. CTCF is an important regulatory factor that controls the expression of genes. As binding sites for CTCF are associated with many repeat sequences, CTCF may play a role in regulating genetic instability in various repeat diseases—not just the one we studied.

## Introduction

Trinucleotide repeat expansion is the cause of at least 25 inherited neurological disorders, including Huntington's disease (HD), fragile X mental retardation, and myotonic dystrophy (DM1) [1]. One intriguing aspect of trinucleotide repeat disorders is ‘anticipation’ – a phenomenon whereby increased disease severity and decreased age-of-onset are observed as the mutation is transmitted through a pedigree [2]. In spinocerebellar ataxia type 7 (SCA7), for example, disease onset in children, who inherit the expanded repeat, averages 20 years earlier than in the affected parent [3]. The basis of the profound anticipation in SCA7 stems from a significant tendency to undergo large repeat expansions upon parent-to-child transmission [4]. Other similarly-sized, disease-linked CAG/CTG repeat tracts do not exhibit strong anticipation, and are much more stable upon intergenerational transmission, as occurs at the spinobulbar muscular atrophy (SBMA) disease locus [5]. Drastic differences in the stability of CAG/CTG repeats, depending upon the locus at which they reside, strongly support the existence of *cis*-acting DNA elements that modulate repeat instability at certain loci. Furthermore, dramatic variation in CAG tract instability in tissues from an individual patient, together with disparities in the timing, pattern, and tissue-selectivity of somatic instability between CAG/CTG disorders, indicates a role for epigenetic modification in DNA instability [1], [6]–9. While the existence of *cis*-elements regulating disease-associated instability is widely accepted, the identities of *cis*-elements that define the mutability of any repeat are still unknown. Proposed *cis*-elements that regulate repeat instability include: the sequence of the repeat tract, the length and purity of the repeat tract, flanking DNA sequences, surrounding epigenetic environment, replication origin determinants, *trans*-factor binding sites, and transcriptional activity [10]–[12]. Such *cis*-elements may enhance or protect against CAG tract instability.

To identify *cis*-elements responsible for CAG expansion at the SCA7 locus, we previously introduced SCA7 CAG-92 repeat expansions into mice, either on 13.5 kb ataxin-7 genomic fragments or on ataxin-7 cDNAs. Comparison of CAG repeat length change revealed that ataxin-7 genomic context drives repeat instability with an obvious bias toward expansion, while SCA7 CAG repeats introduced on ataxin-7 cDNAs were stable [13]. To localize the *cis*-acting elements responsible for this instability tendency, we derived lines of transgenic mice based upon the original 13.5 kb ataxin-7 genomic fragment, deleting a large region (∼8.3 kb) of human sequence beyond the 3′ end of the CAG tract (α-SCA7-92R construct). As deletion of the 3′ region in the α-SCA7-92R transgenic mice significantly stabilized the CAG-92 tract [13], we hypothesized that *cis*-elements within this 3′ region modify repeat instability at the SCA7 locus. To identify *cis*-acting instability elements at the SCA7 locus and the *trans*-acting proteins that regulate them, we evaluated the critical genomic region 3′ to the CAG repeat for sequences that might regulate genetic instability. In the case of SCA7 and a number of other highly unstable CAG/CTG repeat loci, including HD, DM1, SCA2, and dentatorubral-pallidoluysian atrophy, binding sites for a protein known as CTCF (i.e. the “CCCTC binding factor”) have been found [14]. CTCF is an evolutionarily conserved zinc-finger DNA binding protein with activity in chromatin insulation, transcriptional regulation, and genomic imprinting [15],[16]. As CTCF affects higher order chromatin structure [17],[18], we wondered if CTCF binding at the SCA7 locus might regulate CAG repeat instability. To test this hypothesis, we derived SCA7 genomic fragment transgenic mice with CTCF binding site mutations, and found that impaired CTCF binding yielded increases in both intergenerational and somatic instability at the SCA7 locus. Detection of increased somatic instability in association with hypermethylation of the CTCF binding site indicated a role for epigenetic regulation of SCA7 CAG repeat stability. Our results identify CTCF as an important modifier of repeat instability in SCA7, and suggest that CTCF binding may influence repeat instability at other tandem repeat expansion disease loci.

## Results

**Figure 1:**
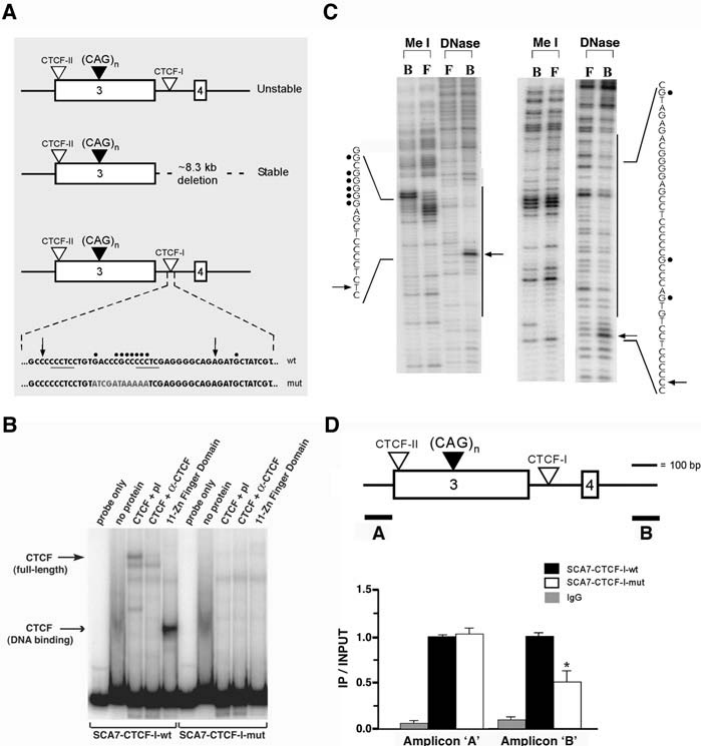
Analysis and mutagenesis of the *SCA7-CTCF-I* binding site. (A) SCA7 genomic fragments used for transgenesis. Upper: *SCA7-CTCF-I*-wt; Middle: α-SCA7 3′ genomic deletion; Bottom: *SCA7-CTCF-I*-mut. Core CCCTC sequences are underlined, and sequence alterations in the *SCA7-CTCF-I*-mut transgenic construct are shown in gray. (B) Electrophoretic mobility shift assays with *SCA7-CTCF-I*-wt and -mut probe fragments were performed with probe only, empty lysate (no protein), full-length CTCF protein with pre-immune anti-CTCF sera (CTCF+pI), CTCF protein with anti-CTCF sera (CTCF+α-CTCF), or the 11 zinc-finger DNA binding domain region of CTCF. Arrows indicate shifted CTCF-DNA complexes. Addition of CTCF-DM1 probe as cold competitor prevented CTCF-DNA complex formation for *SCA7-CTCF-I*-wt fragment, while non-specific cold competitor did not (data not shown). (C) Methylation interference (Me I) and DNase I footprinting (DNase) on *SCA7-CTCF-I* fragment. Left and right panels correspond to the 5′-end labeled coding and anti-sense strands respectively. B, CTCF-bound DNA; F, free DNA; long bars, CTCF-protected from DNase I; arrows, DNase I hypersensitive sites created by CTCF binding; filled circles, contact guanine nucleotides essential for sequence recognition by CTCF. See panel ‘A’ for precise location of sites. (D) ChIP on cerebellar lysates from *SCA7-CTCF-I*-wt and -mut mice (n = 3/genotype). Significantly decreased occupancy at the CTCF-I site was detected with the 3′ amplicon (primer set B) in *SCA7-CTCF-I*-mut mice (p = 0.02, one-way ANOVA), as this amplicon is not in close proximity to the 5′ CTCF-II site. No differences in CTCF occupancy between *SCA7-CTCF-I*-wt and -mut mice were detected with primer set A (or other adjacent primer sets; data not shown) due to the close proximity of the two CTCF binding sites. Results are normalized to *SCA7-CTCF-I*-wt. Error bars are s.d.

At the SCA7 locus, there are two CTCF binding sites that flank the CAG repeat tract; the CTCF-I binding site is located 3′ to the CAG repeat (Figure S1), within the critical region deleted from the SCA7 genomic fragment in the α-SCA7-92R mice (Figure 1A). As CTCF binding sites are associated with highly unstable repeat loci [14], and CTCF binding can alter chromatin structure and DNA conformation [17],[18], we hypothesized that CTCF binding might be involved in SCA7 repeat instability. To test this hypothesis, we decided to compare SCA7 CAG repeat instability in mice carrying either the wild-type CTCF binding site or a mutant CTCF binding site that would be incapable of binding CTCF. To define the CTCF binding sites, we performed electrophoretic mobility shift assays to confirm that CTCF protein specifically binds to the putative CTCF-I binding site, and we found that both the CTCF DNA binding domain fragment and full-length CTCF protein bind to the SCA7 repeat locus 3′ region (Figure 1B). When we mapped the CTCF-I contact regions at the SCA7 repeat locus by methylation interference and DNA footprinting, we defined a region that is protected from DNase I treatment upon CTCF binding and subject to altered CTCF binding upon methylation treatment (Figure 1C). We then introduced point mutations at 11 nucleotides within this 3′ CTCF-I binding site, including eight contact nucleotides contained within the footprinted region (Figure 1C; Figure 1A, bottom). After confirming that CTCF binding was abrogated by these point mutations in electrophoretic mobility shift assays (Figure 1B), we derived a RL-SCA7 94R 13.5 kb genomic fragment construct, that was identical to our original RL-SCA7 92R genomic fragment construct [13], except for: i) the presence of a mutant CTCF-I binding site, and ii) a minor repeat size increase to 94 CAG repeats. The RL-SCA7 94R CTCF-I-mutant construct was microinjected, and two independent lines of RL-SCA7 94R CTCF-I mutant transgenic mice were generated (hereafter referred to as the ***SCA7-CTCF-I*** **-mut** line mice – to distinguish them from the original RL-SCA7-92R transgenic mice with an intact CTCF-I binding site, hereafter referred to as the ***SCA7-CTCF-I*** **-wt** line mice).

To assess *in vivo* occupancy of the CTCF-I binding site in *SCA7-CTCF-I*-wt and *SCA7-CTCF-I*-mut mice, we performed chromatin immunoprecipitation (ChIP) assays. To distinguish between the two CTCF binding sites, separated by a distance of 562 bp, we used two primer sets, including one extending 3′ to the CAG repeat. Quantitative PCR amplification with a primer set (‘A’) within ∼800 bp of the CTCF-I and CTCF-II sites yielded comparable CTCF occupancy in *SCA7-CTCF-I*-wt and -mut mice. As most sheared DNA fragments isolated by ChIP exceed 1 kb, intact CTCF-II sites and the primer set ‘A’ amplicon will be present in sheared DNA fragments isolated by ChIP from *SCA7-CTCF-I*-wt and -mut mice, accounting for comparable CTCF occupancy with primer set A. However, a significant reduction in CTCF occupancy at the CTCF-I site was observed in the *SCA7-CTCF-I*-mut mice for primer set B, which is closer to the CTCF-I binding site (at a distance of ∼700 bp) than the CTCF-II binding site (at a distance of ∼1,200 bp, thereby exceeding the size of most sheared DNA fragments isolated by ChIP) (Figure 1D; p = 0.02, one-way ANOVA). Thus, ChIP analysis indicated that *in vivo* CTCF-I occupancy is significantly diminished in the cerebellum of *SCA7-CTCF-I*-mut mice.

**Figure 2:**
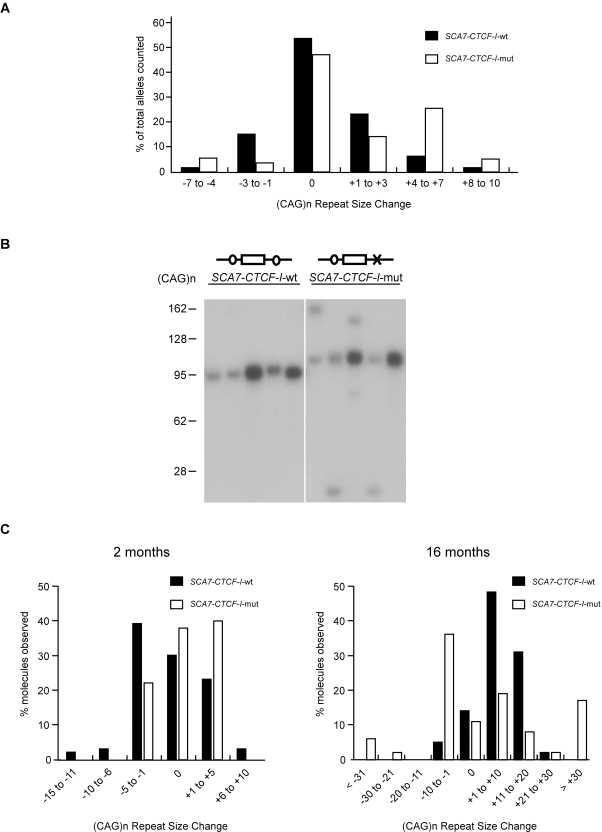
*SCA7-CTCF-I*-mut mice display increased germ line instability. (A) Comparison of CAG repeat instability in parent-offspring transmissions for *SCA7-CTCF-I* mice. Repeat lengths are plotted as % of total alleles scored for 53 *SCA7-CTCF-I*-wt and 95 *SCA7-CTCF-I*-mut mice. The repeat size range in the *SCA7-CTCF-I*-mut mice was significantly different from the distribution of repeat alleles in the *SCA7-CTCF-I*-wt mice (p = 0.002; Mann-Whitney two-tailed test). (B) Small-pool PCR of sperm DNAs in 16 month-old SCA7 transgenic mice. *SCA7-CTCF-I*-wt mice typically exhibited small repeat length changes, while *SCA7-CTCF-I*-mut mice displayed pronounced instability. (C) Compilation of small-pool PCR data. At 2 months of age, only modest instability was noted. At 16 months of age, *SCA7-CTCF-I*-wt mice displayed moderate instability, but *SCA7-CTCF-I*-mut mice exhibited significantly greater instability (p = 0.009; Mann-Whitney two-tailed test).

We assessed intergenerational repeat length instability in 3 month-old *SCA7-CTCF-I*-wt and *SCA7-CTCF-I*-mut mice by PCR amplification of the CAG repeat from tail DNAs, and found that mutation of the CTCF-I site destabilized the CAG repeat during intergenerational transmission (p = 0.002, Mann-Whitney two-tailed test) (Figure 2A). Increased intergenerational instability in the *SCA7-CTCF-I*-mut mice was reflected by a broader range of repeat length change, as mean expansion and deletion sizes were greater for *SCA7-CTCF-I*-mut mice in comparison to *SCA7-CTCF-I*-wt mice (+4.4 CAG's/−4.7 CAG's vs. +2.6 CAG's/−2.0 CAG's). Analysis of repeat length instability between the two *SCA7-CTCF-I-*mut lines revealed similar intergenerational repeat instability (p = 0.93, chi-square), and there was no difference in expansion bias between the two lines (p = 0.25, chi-square). Thus, the *SCA7-CTCF-I-*mut mice did not show integration site effects, suggesting that increased instability in the two lineages results from altered CTCF binding. We then assessed germ line repeat instability by small-pool PCR of individual alleles in sperm DNAs from mice at age 2 months and 16 months (Figure 2B–C). As the mice aged, the CAG repeat in *SCA7-CTCF-I*-mut mice became increasingly unstable (p = 0.009, Mann-Whitney two-tailed test), as mean expansion and deletion sizes were significantly greater for 16 month-old *SCA7-CTCF-I*-mut mice in comparison to *SCA7-CTCF-I*-wt mice (+24.3 CAG's/−15.5 CAG's (mut) vs. +9.2 CAG's/−1.0 CAG (wt)). Increasing CAG repeat instability with aging in *SCA7-CTCF-I*-mut mice suggests a role for CTCF in DNA instability during spermatogenesis, or for the male germ line-restricted CTCF-like paralogue (CTCFL), also known as brother of the regulator of imprinted sites, or ‘BORIS’ [19]. A potential role for CTCFL/BORIS in male germ line instability in the *SCA7-CTCF-I*-mut mice is plausible, as mutation of the *SCA7-CTCF*-I site also prevented binding of CTCFL/BORIS in electrophoretic mobility shift assays (Figure S2).

**Figure 3:**
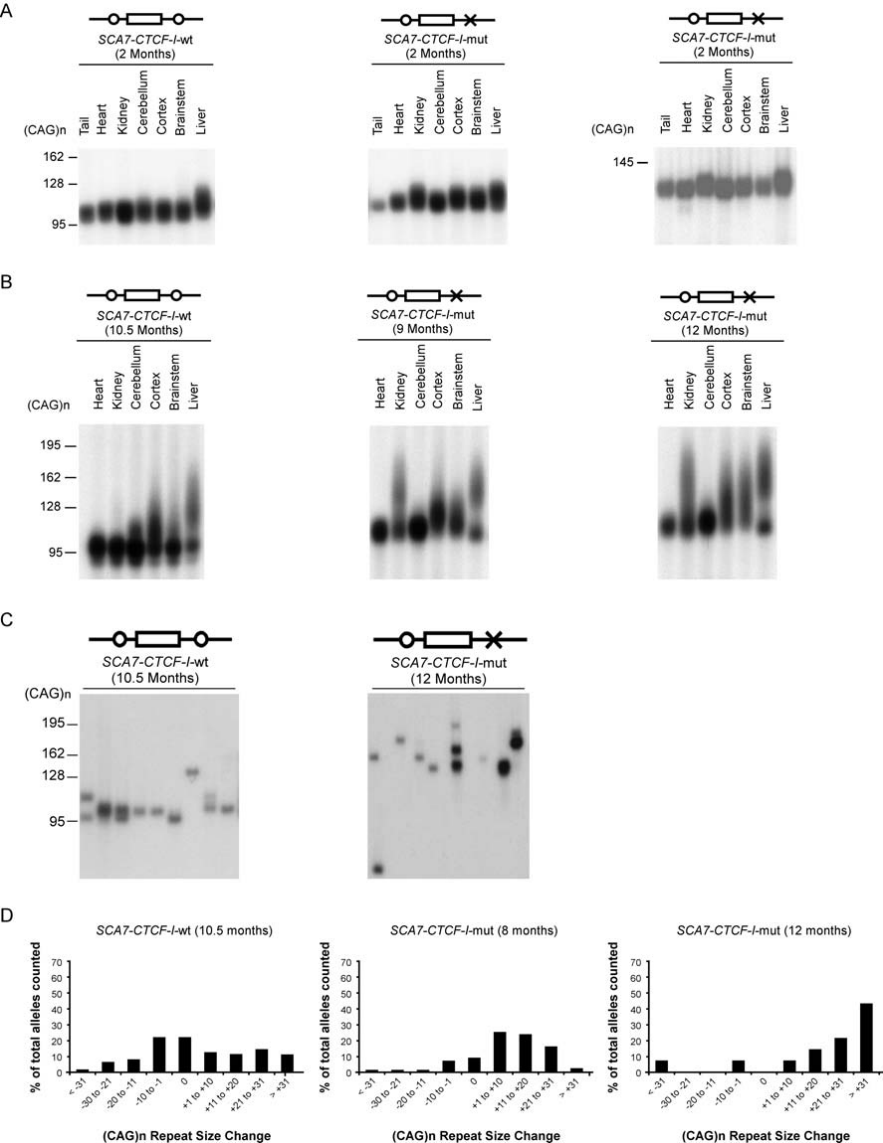
*SCA7-CTCF-I*-mut mice display increased somatic instability. (A) At 2 months of age, the SCA7 CAG repeat is stable in the *SCA7-CTCF-I*-wt line and in both *SCA7-CTCF-I*-mut lines. (B) With advancing age, tissue-specific instability is seen in *SCA7-CTCF-I*-wt mice; however, this tissue-specific instability is much more pronounced in *SCA7-CTCF-I*-mut mice. Results for individuals from the two different *SCA7-CTCF-I*-mut mice are shown here. (C) To permit quantification of somatic instability, we performed small-pool PCR on tissue DNA samples from *SCA7-CTCF-I*-wt and *SCA7-CTCF-I*-mut mice. As shown here for cortex, *SCA7-CTCF-I*-mut mice displayed significantly greater instability than *SCA7-CTCF-I*-wt mice (p = 8.6×10^−5^, Mann-Whitney two-tailed test). See Table 1 for a compiled list of repeat alleles. (D) Histogram of repeat length variation in the cortex of *SCA7-CTCF-I*-wt and *SCA7-CTCF-I*-mut mice. *SCA7-CTCF-I*-mut mice exhibit significantly greater instability than *SCA7-CTCF-I*-wt mice, and this expansion tendency exceeds that of *SCA7-CTCF-I*-wt mice, even when 2.5 months younger (p = 0.0003, Mann-Whitney two-tailed test). With advancing age, the expansion bias between the *SCA7-CTCF-I*-mut and -wt mice becomes more pronounced (p<.0001, Mann-Whitney two-tailed test). Results for individuals from the two different *SCA7-CTCF-I*-mut mice are shown here.

Another intriguing feature of repeat instability is variation in repeat size within and between the tissues of an individual organism. This tissue-specific instability, or “somatic mosaicism”, occurs in human patients with repeat diseases, and in mouse models of repeat instability and disease [1],[8],[11]. While shown to be age-dependent, the mechanistic basis of inter-tissue variation, which even occurs in postmitotic neurons [20], is unknown. To determine if somatic CAG mosaicism at the SCA7 locus involves CTCF binding, we surveyed repeat instability in various tissues from *SCA7-CTCF-I*-wt and *SCA7-CTCF-I*-mut mice. At two months of age, the SCA7 CAG repeat was remarkably stable in all analyzed tissues (Figure 3A). However, by ∼10 months of age, *SCA7-CTCF-I*-wt and *SCA7-CTCF-I*-mut mice displayed large CAG repeat expansions in the cortex and liver (Figure 3B). The liver also exhibited a bimodal distribution of repeat size (i.e. two populations of cells with distinct tract lengths) (Figure 3B). The most pronounced somatic instability differences existed in the kidney, with large expansions for *SCA7-CTCF-I*-mut mice, but stable repeats in the *SCA7-CTCF-I*-wt mice (Figure 3B). This pattern of increased kidney and liver repeat instability was present in both *SCA7-CTCF-I*-mut transgenic lines (Figure 3B; Figure S3). Indeed, comparable somatic instability was also detected in both *SCA7-CTCF-I*-mut transgenic lines at five months of age (Figure S4). When we closely examined repeat instability in the cortex by small-pool PCR, we observed significantly different repeat sizes (p = 8.6×10^−5^, Mann-Whitney), with a range of 39 to 152 CAG repeats in *SCA7-CTCF-I*-wt mice and 26 to 245 CAG repeats in *SCA7-CTCF-I*-mut mice (Figure 3C; Table 1). The increased somatic instability occurred in both *SCA7-CTCF-I*-mut transgenic lines, as an expansion bias was apparent in both lineages upon small-pool PCR analysis (Figure 3D; Table 1). These findings suggest that CTCF binding stabilizes the SCA7 CAG repeat in certain tissues. Thus, as noted for the germ line and documented for two independent lines of *SCA7-CTCF-I*-mut transgenic mice, SCA7 somatic CAG instability is dependent upon age and the presence of intact CTCF binding sites.

**Table 1:** Repeat sizes of cortex DNA: CAG tract length - Small-pool PCR.

*SCA7-CTCF-I-wt*		*SCA7-CTCF-I-mut*		*SCA7-CTCF-I-mut*		*SCA7-CTCF-I-mut*
(10.5 months)		(2 months)		(8 months) *		(12 months) *
39	98	91	103	2	109	26
78	98	91	103	75	109	36
83	101	91	110	79	109	111
83	101	91	130	90	109	119
83	104	92		90	112	129
83	104	92		91	112	129
86	107	95		95	112	134
86	107	95		95	112	134
86	107	95		96	112	134
86	111	95		96	112	140
86	114	95		96	116	144
86	114	96		96	118	144
86	114	96		96	118	145
86	117	96		96	120	145
89	117	96		99	120	148
92	117	96		100	120	150
92	117	96		100	120	152
92	121	96		100	120	154
92	121	96		100	122	158
92	121	96		103	125	160
95	121	98		103	125	162
95	124	98		103	125	165
95	128	98		103	129	165
95	136	98		103	131	172
95	136	99		103	131	177
95	136	99		103	131	177
95	139	99		104	138	199
95	144	99		104	152	245
95	152	99		104	155	
95		101		104	176	
95		101		104		
95		101		107		
95		103		107		
95		103		107		
98		103		107		
98		103		107		

*: Results for individuals from the two different *SCA7-CTCF-I*-mut lines are shown here.

**Figure 4:**
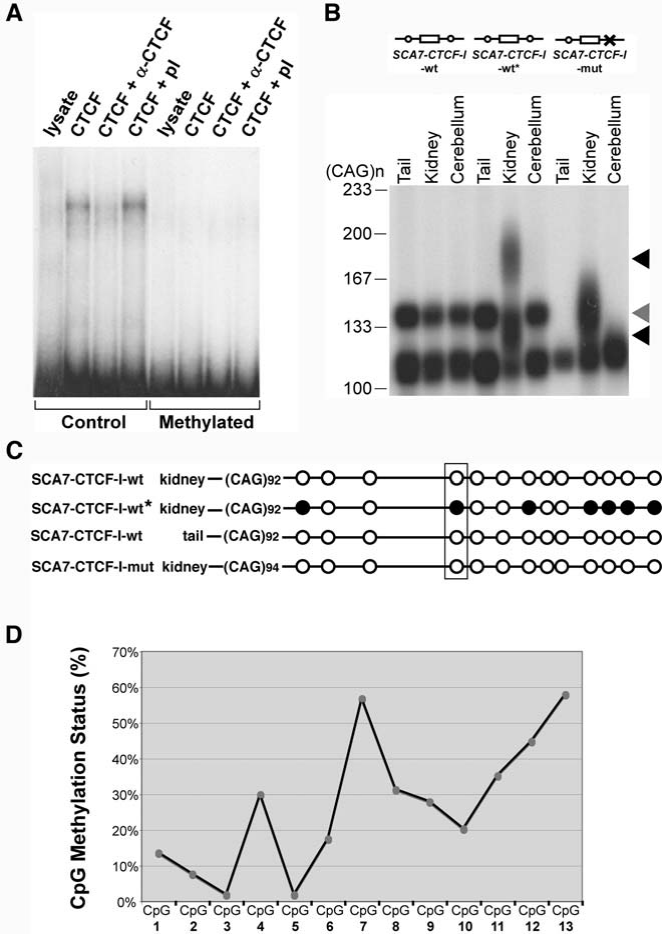
Epigenetic regulation of CTCF binding modulates instability at the SCA7 locus. (A) CpG methylation prevents binding of CTCF to *SCA7-CTCF-I* site. Electrophoretic mobility shift assays with un-methylated (control) or methylated *SCA7-CTCF-I* fragments, using CTCF with no antisera (CTCF), CTCF with anti-CTCF antisera (CTCF+α-CTCF), or CTCF with pre-immune sera (CTCF+pI). Arrow indicates CTCF-bound probe. (B) Prominent somatic instability in kidney DNA (black arrowheads) from a *SCA7-CTCF-I*-wt mouse with CTCF-I site methylation (*SCA7-CTCF-I*-wt*) contrasts with somatic stability in *SCA7-CTCF-I*-wt mice with un-methylated CTCF-I sites. Note that *SCA7-CTCF-I*-wt lines display bimodal CAG repeat alleles. Prominent somatic instability is apparent in kidney DNA (gray arrowhead) from a *SCA7-CTCF-I*-mut mouse. All mice were 6 months of age. (C) Kidney DNAs from the *SCA7-CTCF-I*-wt* mouse are highly methylated. Circles, CpG dyads; open circles, unmethylated; filled circles; methylated. Box highlights core CTCF binding site contact residue, based upon footprinting analysis. Diagrammed epigenotypes summarize results for five *SCA7-CTCF-I*-wt mice, eight *SCA7-CTCF-I*-mut mice, and the *SCA7-CTCF-I*-wt* mouse, and were consistent for at least 75% of all sequenced clones (n = 10−12/sample). (D) Liver DNAs from control *SCA7-CTCF-I*-wt mice are methylated. Bisulfite sequencing of the *SCA7-CTCF-I* region was performed upon liver DNAs from three *SCA7-CTCF-I*-wt mice at one year of age (n = 17 clones/mouse), and CpG methylation determined for the 13 CpG dyads in the *SCA7-CTCF-I* region. A number of CpG dyads, including the CpG-4 CTCF contact site, exhibit moderate to high levels of methylation.

CTCF binding can be regulated by CpG methylation, as methylation at CTCF recognition sites abrogates binding [16]. This finding was confirmed for un-methylated and methylated versions of the SCA7 CTCF-I recognition site (Figure 4A; Figure S5). Highly variable levels of instability have been documented in the kidneys of transgenic repeat instability mouse models [21],[22], although the reasons for pronounced instability in this tissue are unknown. Interestingly, one mouse with a wild-type CTCF-I binding site (*SCA7-CTCF-I*-wt) displayed marked CAG repeat instability in its kidney DNA (Figure 4B), paralleling the considerable instability observed in the *SCA7-CTCF-I*-mut mice (Figure 3B). Bisulfite sequencing of kidney DNA from this *SCA7-CTCF-I*-wt mouse revealed high levels of CpG methylation at the wild-type CTCF-I binding site, including the central CTCF contact site (Figure S6); whereas methylation was not observed in kidney DNAs from 14 other *SCA7-CTCF-I-wt* mice that displayed only modest levels of CAG instability (Figure 4C). The high levels of CAG instability and the CpG methylation in this mouse were restricted to the kidney, as the cerebellum and tail DNAs of the same mouse, which showed limited CAG instability (Figure 4B), were completely unmethylated (Figure 4C). This finding suggests a direct link between methylation status of the CTCF binding site and CAG repeat instability. Of all the tissues analyzed from *SCA7-CTCF-I-*wt mice, liver exhibits the greatest amount of somatic mosaisicm, with the largest repeat expansions (Figure 3B). We hypothesized that the high levels of CAG repeat instability in the liver of *SCA7-CTCF-I*-wt mice might result from methylation of the CTCF-I binding site. To address this question, we performed bisulfite sequencing analysis of liver DNAs from *SCA7-CTCF-I-*wt mice, and documented moderately high levels of methylation at the CTCF-I binding site (Figure 4D; Figure S7). These results indicate a correlation between CpG methylation and CAG repeat instability. Thus, in SCA7 transgenic mice, decreased CTCF binding, either by CpG methylation or mutagenesis of the CTCF-I binding site, enhanced CAG repeat instability.

## Discussion

**Figure 5:**
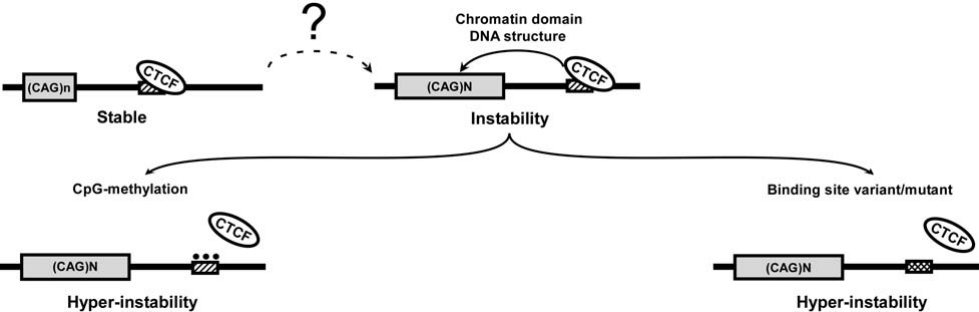
Model for CTCF regulation of CAG repeat instability. Non-expanded CAG repeat is stable, as CTCF is bound to adjacent site. Upon repeat expansion, chromatin environment and DNA structure of repeat region is altered, permitting instability. Loss of CTCF binding at adjacent CTCF binding site, either by CpG methylation or CTCF binding site mutation, further promotes repeat instability.

We have identified a CTCF binding site as the first *cis*-element regulating CAG tract instability at a disease locus. Furthermore, binding of the *trans*-factor CTCF to this *cis*-element influences CAG instability, and this interaction is epigenetically regulated. At the SCA7 locus and four other CAG/CTG repeat loci known to display pronounced anticipation, functional CTCF binding sites occur immediately adjacent to the repeats, and CTCF binding can affect DNA structure and chromatin packaging at such loci, and elsewhere [14], [23]–[26]. Although an interplay between GC-content, CpG islands, epigenetic modification, chromatin structure, repeat length, and unusual DNA conformation has long been postulated to underlie trinucleotide repeat instability [11], [27]–[29], the mechanistic basis of this process is ill-defined. CTCF insulator and genomic imprinting functions are subject to epigenetic regulation, as methylation status is a key determinant of CTCF action at certain “differentially methylated domains” and methylation changes at CTCF binding sites are linked to oncogenic transformation [16],[18]. At the SCA7 locus, methylation status of the CTCF-I binding site may be similarly important for its ability to tamp down repeat instability, as hypermethylation of the CTCF-I site was associated with a dramatic enhancement of somatic instability in the SCA7 genomic fragment transgenic mouse model. Thus, inability to bind CTCF at sites adjacent to CAG tracts, because of binding site mutation or CpG methylation in the case of the *SCA7-CTCF-I* site, can promote further expansion of disease-length CAG repeat alleles (Figure 5).

In both human patients and transgenic mice with expanded repeat tracts, the repeat displays high levels of instability. The flanking sequence has been thought to contain elements that may protect or enhance repeat instability. Our results show that CTCF binding is a stabilizing force at the SCA7 repeat locus, suppressing expansion of the CAG repeat in the germ line and soma. Interestingly, deletion of ∼8.3 kb of 3′ genomic sequence in our previous SCA7 transgenic mouse, including the CTCF-I site, stabilized the repeat [13]. The CAG-92 stabilization, arising from the ∼8.3 kb 3′ genomic fragment deletion, suggests the existence of positive *cis*-regulators that were “driving” CAG instability. One such element could be a replication initiation site that was mapped within the genomic region 3′ to the CTCF-I binding site at the SCA7 locus [30]. Hence, the 8.3 kb 3′ deletion could grossly alter the chromatin organization of the adjacent repeat, and would likely ablate replication origin activity, stabilizing the CAG repeat tract. However, this ∼8.3 kb genomic region likely also contained negative *cis*-regulators of CAG repeat instability, whose dampening effects would not be apparent due to the coincident loss of instability drivers. Our results indicate that CTCF binding negatively regulates expanded CAG repeat instability at the SCA7 locus. CTCF regulation of repeat instability potential is consistent with its many roles in modulating DNA structure. CTCF can mediate long-range chromatin interactions and can co-localize physically distant genomic regions into discrete sub-nuclear domains [17],[18]. CTCF insulates heterochromatin and silenced genes from transcriptionally active genes, as CTCF binding sites occur at transition zones between X-inactivation regions and genes that escape from X-inactivation [24]. CTCF has been implicated in genomic imprinting, although recent studies indicate that such transcription insulator events may involve the coordinated action of CTCF with cohesin [31]–[33]. CTCF binding at the DM1 locus sequesters repeat-driven heterochromatin formation to the immediate repeat region, while repeat expansion-induced loss of CTCF binding may permit spreading of heterochromatin to adjacent genes, accounting for the mental retardation phenotype in congenital DM1 [23]. As DNA structural conformation and transcription activity are two highly intertwined processes that appear fundamental to the instability of expanded tandem repeats [10],[11], CTCF appears a likely candidate for modulation of trinucleotide repeat instability.

At the SCA7 locus, a pronounced tendency for repeat expansion has been associated with transmission through the male germ line [3],[4],[34]. Although we have hypothesized that CTCF is principally responsible for modulating SCA7 CAG repeat instability both in the germ line and in the soma, we considered a possible role for the related CTCF-like factor BORIS. BORIS and CTCF share identical 11 zinc-finger domains for DNA binding [19]; hence, both CTCF and BORIS can bind to the CTCF binding sites at the SCA7 locus. Upon mutation or methylation of the CTCF binding site 3′ to the SCA7 CAG repeat, neither CTCF nor BORIS can bind (Figure 1C; Figure 4A; Figure S8). As BORIS can bind to the *H19* differentially methylated domain even when it is methylated [35], our results suggest that the methylation dependence of BORIS binding is locus specific. BORIS and CTCF expression patterns overlap very little, if at all, and in the male germ line, BORIS appears restricted to primary spermatocytes, while CTCF occurs almost exclusively in post-meiotic cells, such as round spermatids [19]. Interestingly, neither BORIS nor CTCF could be detected by immunostaining proliferating spermatogonia. In human HD patients and transgenic mouse models of CTG/CAG instability, large repeat expansions have been documented in spermatogonia, but not in post-meiotic spermatids or spermatozoa [36]–[39]. Thus, absence or low levels of BORIS or CTCF in spermatogonia — the cells in which the largest and most frequent repeat expansions occur — may contribute to the paternal parent-of-origin expansion bias common to most CAG/CTG repeat diseases. In spermatocytes, BORIS may stabilize expanded CAG repeats, just as CTCF binding appears to promote repeat stability in somatic tissues. Thus, in the *SCA7-CTCF-I*-mut mice, abrogated binding of BORIS may contribute to increased repeat instability and expansion bias in the male germ line.

Our findings suggest that CTCF is a *trans*-acting factor that specifically interacts in a methylation-dependent manner with the adjacent *cis*-environment to prevent hyper-expansion of disease length CAG repeats. In a *Drosophila* model of polyglutamine repeat disease, expression of the mutant gene product modulated repeat instability by altering transcription and repair pathways [10]. Similarly, uninterrupted repeat sequences, and in particular, runs of CG-rich trinucleotide repeats, can affect replication machinery, DNA repair pathways, and nucleosome positioning, though in *cis*, by altering the structure and conformation of the DNA regions within which they reside [40],[41]. Association of adjacent CTCF binding sites with repeat loci is a common feature of unstable microsatellite repeats [14]. We propose that acquisition of CTCF binding sites at mutational hot spots represents an evolutionary strategy for insulating noxious DNA sequences [42], and our findings indicate that CTCF binding site utilization at a mutational hot spot is subject to epigenetic regulation. We thus envision a predominant role for CTCF in modulating genetic instability at DNA regions containing variably-sized repeats, unstable sequence motifs, or other repetitive sequence elements.

## Materials and Methods

### Generation of *SCA-CTCF-I*-mut Transgenic Mice

To derive the *SCA7-CTCF-I*-mut transgenic construct, we synthesized a PCR primer with randomly mutated nucleotides introduced at the CTCF-I contact sites for recombineering into the RL-SCA7-92R (*SCA7-CTCF-I*-wt) construct [13], and then confirmed loss of CTCF binding by the mutated fragment by electrophoretic mobility shift assay (protocol provided below). Using a standard recombineering approach [43], we PCR-generated a *SCA7-CTCF-I* targeting cassette containing a Chloramphenicol resistance gene and *Cla* I restriction site flanked by *SCA7-CTCF-I* region sequences with the following primer set: hSCA7-wt-CAM-F, 5′-tcccccctgcccccctcctgtatcgatgtttaagggcaccaataactgc-3′ & hSCA7-mut-CAM-R, 5′-catctctgcccctcga**tttttatcgat**atcgataatgatgagcacttttcgaccg-3′. After recombineering the *SCA7-CTCF-I*-mut targeting cassette into the SCA7-CTCF genomic fragment carried on a plasmid, selection, and PCR screening, we deleted the Chloramphenicol gene by *Cla* I digestion and ligation. We verified the sequence of the *SCA7-CTCF-I*-mut construct prior to linearization with *Sal* I – *Spe* I digestion, gel purification, and microinjection into C57BL/6J×C3H/HeJ oocytes. Transgene-positive founders were backcrossed onto the C57BL/6J background for more than 12 generations to yield incipient congenic mice before repeat instability analysis commenced. All experiments and animal care were performed in accordance with the University of Washington IACUC guidelines.

### Electrophoretic Mobility Shift Assays

We amplified a 161 bp DNA fragment (*SCA7-CTCF-I*) from the SCA7 locus with primers (5′-ctccccccttcaccccctcgagac-3′ & 5′-gtgacgcacactcacgcacgcacgg-3′) labeled at their 5′ ends by γ-^32^P-ATP. We gel-purified the 5′ end-labeled fragment, and used it for electrophoretic mobility shift assays, with *in vitro* translated proteins, as previously described [14]. We synthesized the CTCF-11 zinc finger (ZF) DNA binding domain, full length CTCF and full length CTCFL/BORIS proteins using the pCITE-11ZF, pCITE-7.1, and pCITE-BORIS expression constructs [14],[19],[44], with the TnT reticulocyte lysate coupled *in vitro* transcription-translation system (Promega). For “super-shifts”, we used an anti-CTCF antibody (Upstate Biotechnology) or anti-BORIS antibody [19],[44]. We methylated the end-labeled *SCA7-CTCF-I* fragment with *Sss* I methyl-transferase (New England Biolabs) in the presence of 0.8 mM S-adenosylmethionine. We confirmed the methylation status by restriction enzyme digestion with *Nru* I, and used unmethylated fragment as a control [14].

### DNase I Footprinting and Methlyation Interference Analysis

We PCR-amplified the *SCA7-CTCF-I* fragment and labeled it at the 5′ end on either the coding or anti-sense strand, incubated the purified probes with CTCF and then partially digested them with DNase I, or partially methylated them at guanine residues with dimethyl sulfate, and then incubated them with CTCF. Details of these protocols, as well as our methods for isolation and analysis of free probe DNA fragments on sequencing gels, have been described [14].

### DNA Methylation Sequencing

Bisulfite treatment of tissue DNAs was done as previously described [45], and PCR primers spanning the *SCA7-CTCF-I* region were designed so that they excluded CpG dinucleotides within the binding region. PCR products were then cloned into a Topo TA vector and sequenced. Sequencing of positive control samples, treated with *Sss* I to methylate all cytosines in CpG dyads, were included in every run, and revealed lack of C to T conversion at all CpG dyads in all control samples analyzed.

### Chromatin Immunoprecipitation (ChIP)

We prepared tissues, cross-linked proteins to DNA, and processed tissue samples essentially as we have done previously [46]. However, we doubled the length of the sonication step, and, prior to immunoprecipitation, we fractionated supernatant DNAs on agarose gels to gauge the extent of shearing. After confirming that the bulk of sheared DNAs migrated in the 500–1,000 bp range, we performed immunoprecipitation with an anti-CTCF antibody (Upstate Biotechnology), as described [14]. DNAs were isolated and then subjected to real-time qPCR analysis with different SCA7 genomic region primer and probe sets (available upon request) on an ABI-7700 sequence detection system. For each CTCF ChIP sample, we normalized SCA7 locus occupancy to a control region of the Myc locus lacking CTCF binding sites [14]. All primer and probe sequence sets are available upon request.

### Repeat Instability Analysis

We PCR-amplified the SCA7 CAG repeat from genomic DNA samples in the presence of 0.1µCi of α-^32^P-ATP, and resolved the radiolabeled PCR products on 1.8% agarose gels [13]. For small-pool PCR, dilution of genomic DNA's, yielding 1–5 genome equivalents, was performed prior to amplification and sizing [4]. In all experiments, at least three mice/genotype, or three samples/time point, were analyzed. All primer sequences are available upon request.

## Supporting Information

Figure S1 Sequence of the SCA7-CTCF region. Primary sequence for the 3′ end of intron 2, all of exon 3, and the 5′ end of intron 3 are shown. Intron sequence is lowercase; exon sequence is uppercase. CTCF binding sites are shown in blue. Note that the CTCF-I binding site is located in intron 3, while the CTCF-II binding site encompasses intron 2 - exon 3 boundary. Start site of translation is underlined in blue, and CAG repeat is shown in red. Mapped contact regions from methylation interference and DNase I footprinting analysis are indicated by filled circles, and DNase I hypersensitivity sites are marked by arrows (see Figure 1C). The primer sequences for generation of the probe fragment for all electrophoretic mobility shift assays are underlined in black.
(0.02 MB PDF)

Figure S2 Mutation of SCA7-CTCF-I site also abrogates binding by BORIS. Electrophoretic mobility shift assays with *SCA7-CTCF-I*-wt and -mut probe fragments were performed with probe only, the 11 zinc-finger DNA binding domain region of CTCF, full-length CTCF protein, full-length BORIS protein, BORIS protein with anti-BORIS sera (BORIS+α-BORIS), or BORIS with pre-immune anti-BORIS sera (BORIS+pI). Arrows indicate shifted CTCF-DNA complexes, shifted BORIS-DNA complexes, and super-shifted BORIS-DNA complexes. Addition of CTCF-DM1 probe as cold competitor prevented CTCF-DNA and BORIS-DNA complex formation for the *SCA7-CTCF-I*-wt fragment, while non-specific cold competitor did not (data not shown).
(0.06 MB PDF)

Figure S3 Increased somatic instability in both *SCA7-CTCF*-I-mut transgenic lines. Here, we see representative results for PCR analysis of somatic repeat instability for aged individuals from each of the two *SCA7-CTCF-I*-mut transgenic lines analyzed in this study. Note that comparable patterns of increased somatic mosaicism are observed in each lineage.
(0.73 MB PDF)

Figure S4 Comparable somatic mosaicism in both *SCA7-CTCF-I*-mut transgenic lines. Here, we see representative images for PCR analysis of somatic repeat instability for 5 month-old individuals from each of the two *SCA7-CTCF-I*-mut transgenic lines analyzed in this study. Note that comparable patterns of increased somatic mosaicism are again observed at this earlier point.
(0.66 MB PDF)

Figure S5 Methylation of SCA7-CTCF-I-wt probe fragment for gel shift analysis. *Sss* I was used to methylate cytosine residues in CpG dyads in the *SCA7-CTCF-I*-wt probe fragment. Digestion of control (unmethylated) and *Sss* I-methylated probe fragments with the methylation-sensitive restriction enzyme *Nru* I revealed complete methylation of *Sss* I-treated *SCA7-CTCF-I*-wt probe fragment.
(0.79 MB PDF)

Figure S6 Amplicon for bisulfite sequencing for epigenotype determination. PCR amplification of bisulfite-converted genomic DNA for the fragment shown here was performed to derive CpG methylation status at the *SCA7-CTCF-I* binding site in murine tissues. Intron sequence is lowercase; exon sequence is uppercase. The *SCA7-CTCF-I* binding site is shown in blue. The thirteen CpG dyads included in the epigenotyping are shown, and the dyad with filled circles corresponds to a critical CTCF contact site, based upon footprinting analysis (see Figure 1C).
(0.03 MB PDF)

Figure S7 Epigenotype data for bisulfite sequencing analysis of the CTCF-I binding site region in *SCA7-CTCF-I*-wt transgenic liver. Results of bisulfite sequencing analysis for liver DNAs obtained from three *SCA7-CTCF-I*-wt transgenic mice reveal moderate to high levels of CpG methylation in this tissue, especially when compared to the completely un-methylated status of CpG dyads observed in all tail DNAs and kidney DNAs, with one exception.
(0.53 MB PDF)

Figure S8 Methylation of the SCA7-CTCF-I site abrogates binding of BORIS as well as CTCF. Gel retardation assays with unmethylated or Sss I-methylated *SCA7-CTCF-I*-wt probe fragments were performed with probe only, the 11 zinc-finger DNA binding domain region of CTCF, CTCF with pre-immune anti-CTCF sera (CTCF+pI), CTCF protein with anti-CTCF sera (CTCF+α-CTCF), BORIS with pre-immune anti-BORIS sera (BORIS+pI), or BORIS protein with anti-BORIS sera (BORIS+α-BORIS). Arrows indicate shifted CTCF-DNA complexes and shifted BORIS-DNA complexes. Methylation of the SCA7-CTCF-I probe fragment abrogates all binding. Success of Sss I methylation was confirmed by Nru I restriction digestion (see Figure S5).
(2.87 MB PDF)
