# Supplementary material for: CTCF cis-Regulates Trinucleotide Repeat Instability in an Epigenetic Manner: A Novel Basis for Mutational Hot Spot Determination
Source: PLoS Genet. 2008 Nov 14;4(11):e1000257. doi: 10.1371/journal.pgen.1000257 (PMC2573955; doi:10.1371/journal.pgen.1000257)
Supplement: Figure S1 — Sequence of the SCA7-CTCF region. Primary sequence for the 3′ end of intron 2, all of exon 3, and the 5′ end of intron 3 are shown. Intron sequence is lowercase; exon sequence is uppercase. CTCF binding sites are shown in blue. Note that the CTCF-I binding site is located in intron 3, while the CTCF-II binding site encompasses intron 2 - exon 3 boundary. Start site of translation is underlined in blue, and CAG repeat is shown in red. Mapped contact regions from methylation interference and DNase I footprinting analysis are indicated by filled circles, and DNase I hypersensitivity sites are marked by arrows (see Figure 1C). The primer sequences for generation of the probe fragment for all electrophoretic mobility shift assays are underlined in black. (0.02 MB PDF) [file pgen.1000257.s001.pdf]

cgaaagctag cccgcgccgc ggactttgag cccggggcgg ggggtggcct

tgaggaggcg ggctcggggg gctgggcggc catgggggcg ctgtcagcgt

gccccacccg gtccgcgggc cgcgcacgcc gccggaactc cctggcgcct **Intron 2**

ccttaaaaaa cggcccccgc gcgactcttt cccccttttt tttgttacat

tgtagGAGCG GAAAGAATGT CGGAGCGGGC CGCGGATGAC GTCAGGGGGG

AGCCGCGCCG CGCGGCGGCG GCGGCGGGCG GAGCAGCGGC CGCGGCCGCC

CGGCAGCAGC AGCAGCAGCA GCAGCAGCAG CAGCCGCCGC CTCCGCAGCC

CCAGCGGCAG CAGCACCCGC CACCGCCGCC ACGGCGCACA CGGCCGGAGG **Exon 3**

ACGGCGGGCC CGGCGCCGCC TCCACCTCGG CCGCCGCAAT GGCGACGGTC

GGGGAGCGCA GGCCTCTGCC CAGTCCTGAA GTGATGCTGG GACAGTCGTG

GAATCTGTGG GTAGAGGCTT CCAAACTTCA TGGGAAGGAC Ggtgagtgtc

cacgccctcc tccccccttc accccctcgc gaccccctcc tctctcctcc

cctcccccct **gcccccctcc tgtgacccgc cccctcgagg ggcagagatg**

ctatcgtttg ctgggttgcg gaacgcggag gtgcccacac ctaccccgtg **Intron 3**

cgtgcgtgag tgtgcgtcac actcctggcc actgacctgc ctctcccctc

ctcctgtgtg tgtatatctc ctagggacag aattggacga aagtttcaag
