# Supplementary figures and images for: CTCF cis-Regulates Trinucleotide Repeat Instability in an Epigenetic Manner: A Novel Basis for Mutational Hot Spot Determination
Source: PLoS Genet. 2008 Nov 14;4(11):e1000257. doi: 10.1371/journal.pgen.1000257 (PMC2573955; doi:10.1371/journal.pgen.1000257)

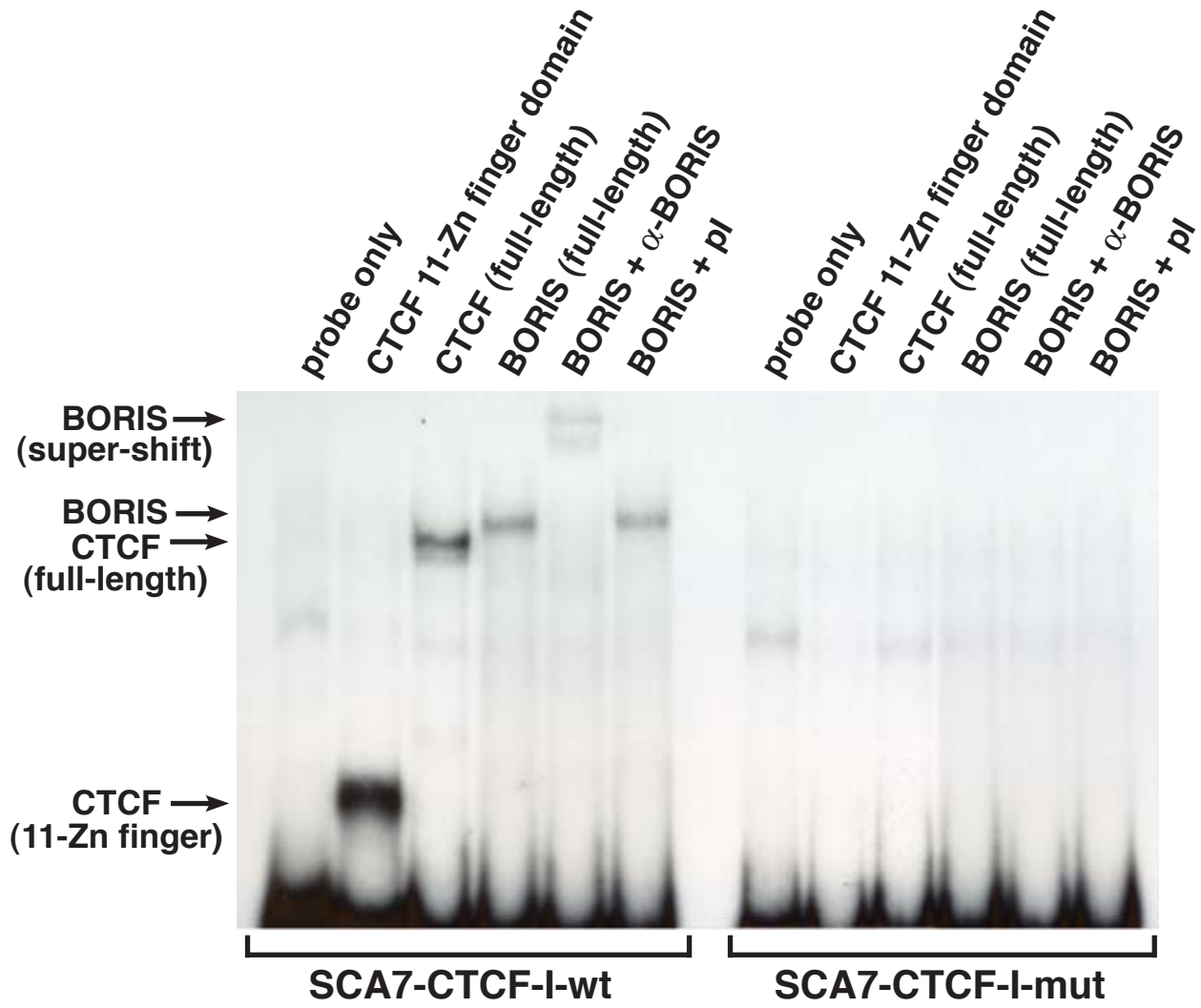

Supplement: Figure S2 — Mutation of SCA7-CTCF-I site also abrogates binding by BORIS. Electrophoretic mobility shift assays with SCA7-CTCF-I-wt and -mut probe fragments were performed with probe only, the 11 zinc-finger DNA binding domain region of CTCF, full-length CTCF protein, full-length BORIS protein, BORIS protein with anti-BORIS sera (BORIS+α-BORIS), or BORIS with pre-immune anti-BORIS sera (BORIS+pI). Arrows indicate shifted CTCF-DNA complexes, shifted BORIS-DNA complexes, and super-shifted BORIS-DNA complexes. Addition of CTCF-DM1 probe as cold competitor prevented CTCF-DNA and BORIS-DNA complex formation for the SCA7-CTCF-I-wt fragment, while non-specific cold competitor did not (data not shown). (0.06 MB PDF) [file pgen.1000257.s002.pdf]

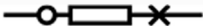

SCA7-CTCF-I-mut

(5 Months)

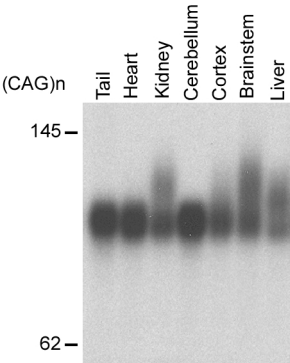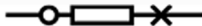

SCA7-CTCF-I-mut

(5 Months)

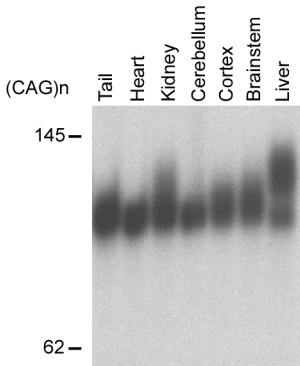

Supplement: Figure S4 — Comparable somatic mosaicism in both SCA7-CTCF-I-mut transgenic lines. Here, we see representative images for PCR analysis of somatic repeat instability for 5 month-old individuals from each of the two SCA7-CTCF-I-mut transgenic lines analyzed in this study. Note that comparable patterns of increased somatic mosaicism are again observed at this earlier point. (0.66 MB PDF) [file pgen.1000257.s004.pdf]

# SCA7-CTCF-I

- Nru I - Nru I

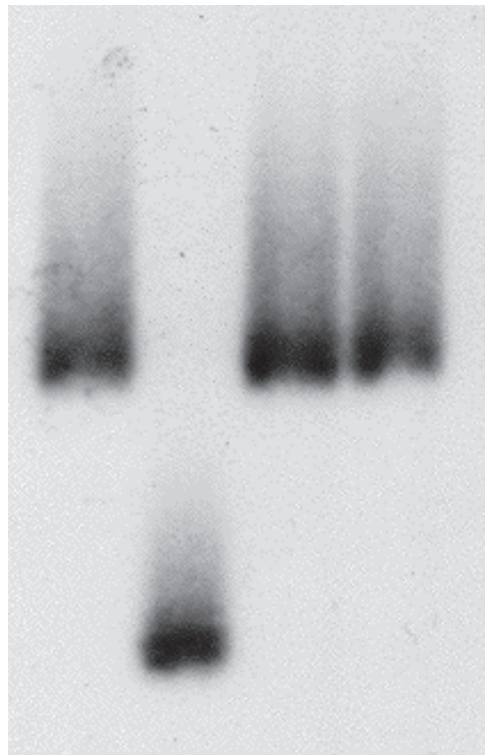

Control Sss I

Supplement: Figure S5 — Methylation of SCA7-CTCF-I-wt probe fragment for gel shift analysis. Sss I was used to methylate cytosine residues in CpG dyads in the SCA7-CTCF-I-wt probe fragment. Digestion of control (unmethylated) and Sss I-methylated probe fragments with the methylation-sensitive restriction enzyme Nru I revealed complete methylation of Sss I-treated SCA7-CTCF-I-wt probe fragment. (0.79 MB PDF) [file pgen.1000257.s005.pdf]

SCA7-CTCF-I-wt (mouse 1)

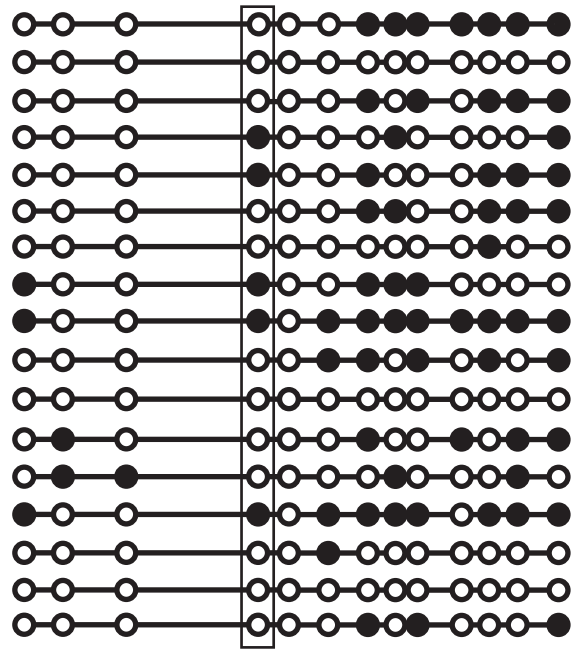

SCA7-CTCF-I-wt (mouse 2)

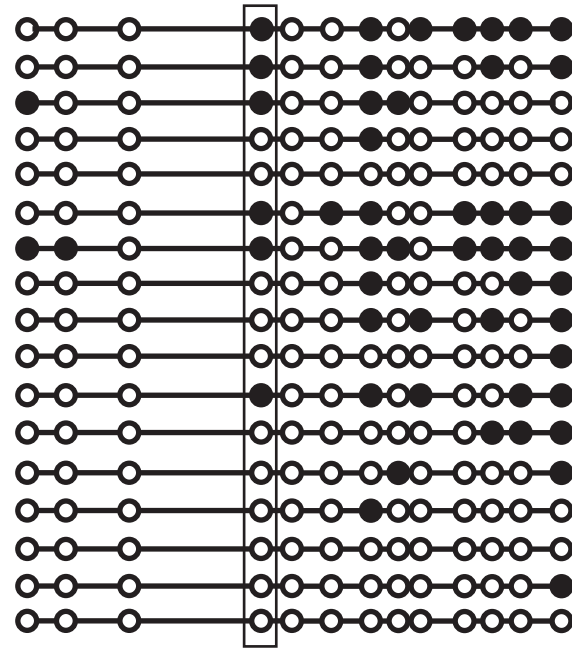

SCA7-CTCF-I-wt (mouse 3)

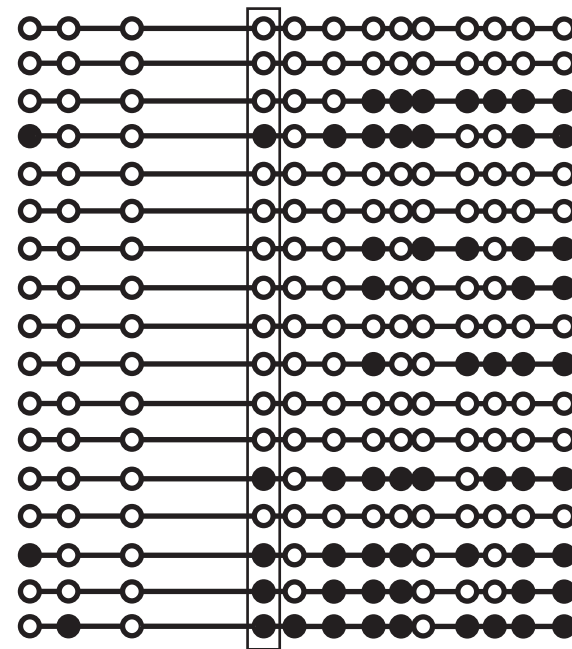

Supplement: Figure S7 — Epigenotype data for bisulfite sequencing analysis of the CTCF-I binding site region in SCA7-CTCF-I-wt transgenic liver. Results of bisulfite sequencing analysis for liver DNAs obtained from three SCA7-CTCF-I-wt transgenic mice reveal moderate to high levels of CpG methylation in this tissue, especially when compared to the completely un-methylated status of CpG dyads observed in all tail DNAs and kidney DNAs, with one exception. (0.53 MB PDF) [file pgen.1000257.s007.pdf]

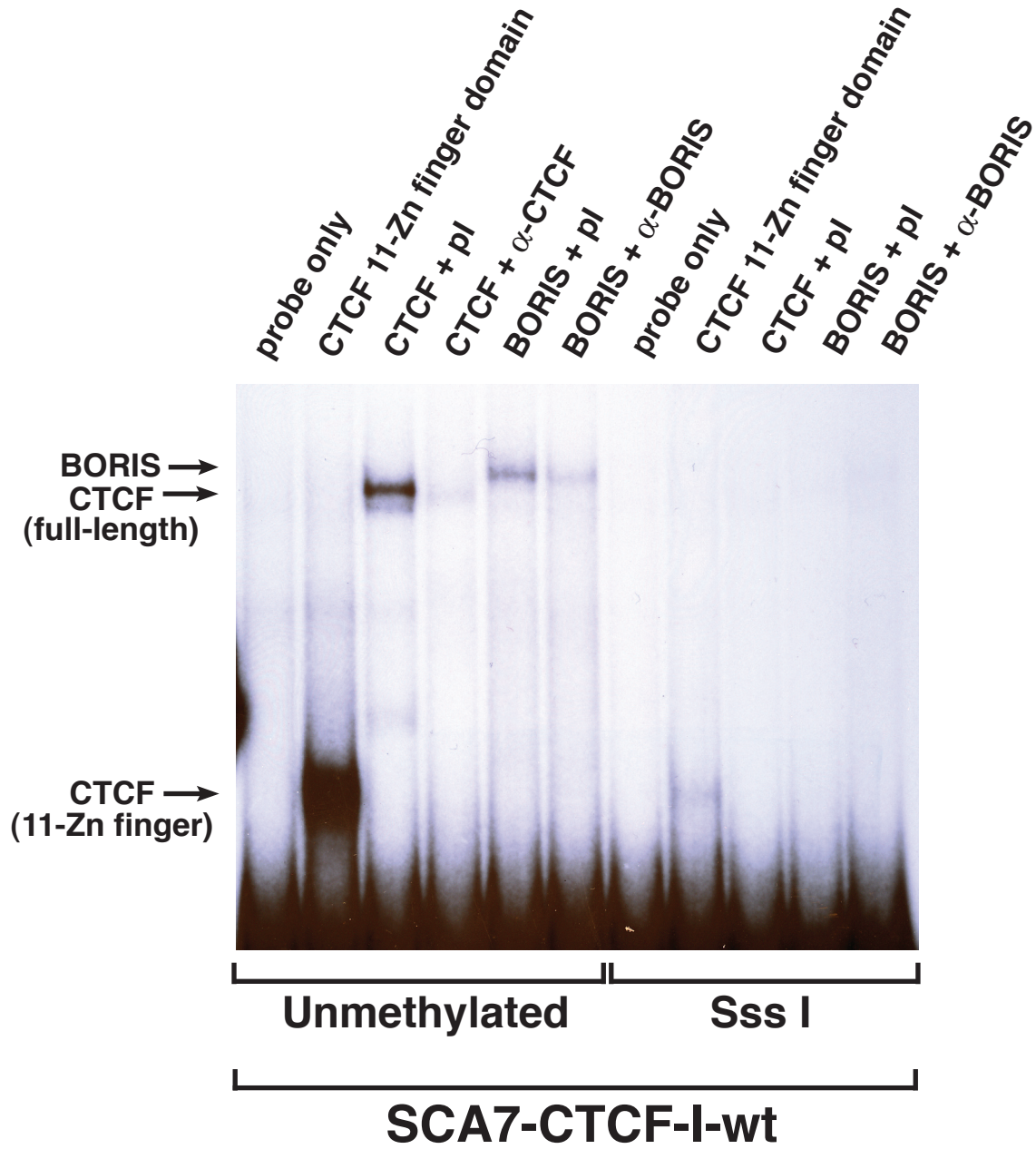

Supplement: Figure S8 — Methylation of the SCA7-CTCF-I site abrogates binding of BORIS as well as CTCF. Gel retardation assays with unmethylated or Sss I-methylated SCA7-CTCF-I-wt probe fragments were performed with probe only, the 11 zinc-finger DNA binding domain region of CTCF, CTCF with pre-immune anti-CTCF sera (CTCF+pI), CTCF protein with anti-CTCF sera (CTCF+α-CTCF), BORIS with pre-immune anti-BORIS sera (BORIS+pI), or BORIS protein with anti-BORIS sera (BORIS+α-BORIS). Arrows indicate shifted CTCF-DNA complexes and shifted BORIS-DNA complexes. Methylation of the SCA7-CTCF-I probe fragment abrogates all binding. Success of Sss I methylation was confirmed by Nru I restriction digestion (see Figure S5). (2.87 MB PDF) [file pgen.1000257.s008.pdf]
