# Supplementary material for: CTCF cis-Regulates Trinucleotide Repeat Instability in an Epigenetic Manner: A Novel Basis for Mutational Hot Spot Determination
Source: PLoS Genet. 2008 Nov 14;4(11):e1000257. doi: 10.1371/journal.pgen.1000257 (PMC2573955; doi:10.1371/journal.pgen.1000257)
Supplement: Figure S3 — Increased somatic instability in both SCA7-CTCF-I-mut transgenic lines. Here, we see representative results for PCR analysis of somatic repeat instability for aged individuals from each of the two SCA7-CTCF-I-mut transgenic lines analyzed in this study. Note that comparable patterns of increased somatic mosaicism are observed in each lineage. (0.73 MB PDF) [file pgen.1000257.s003.pdf]

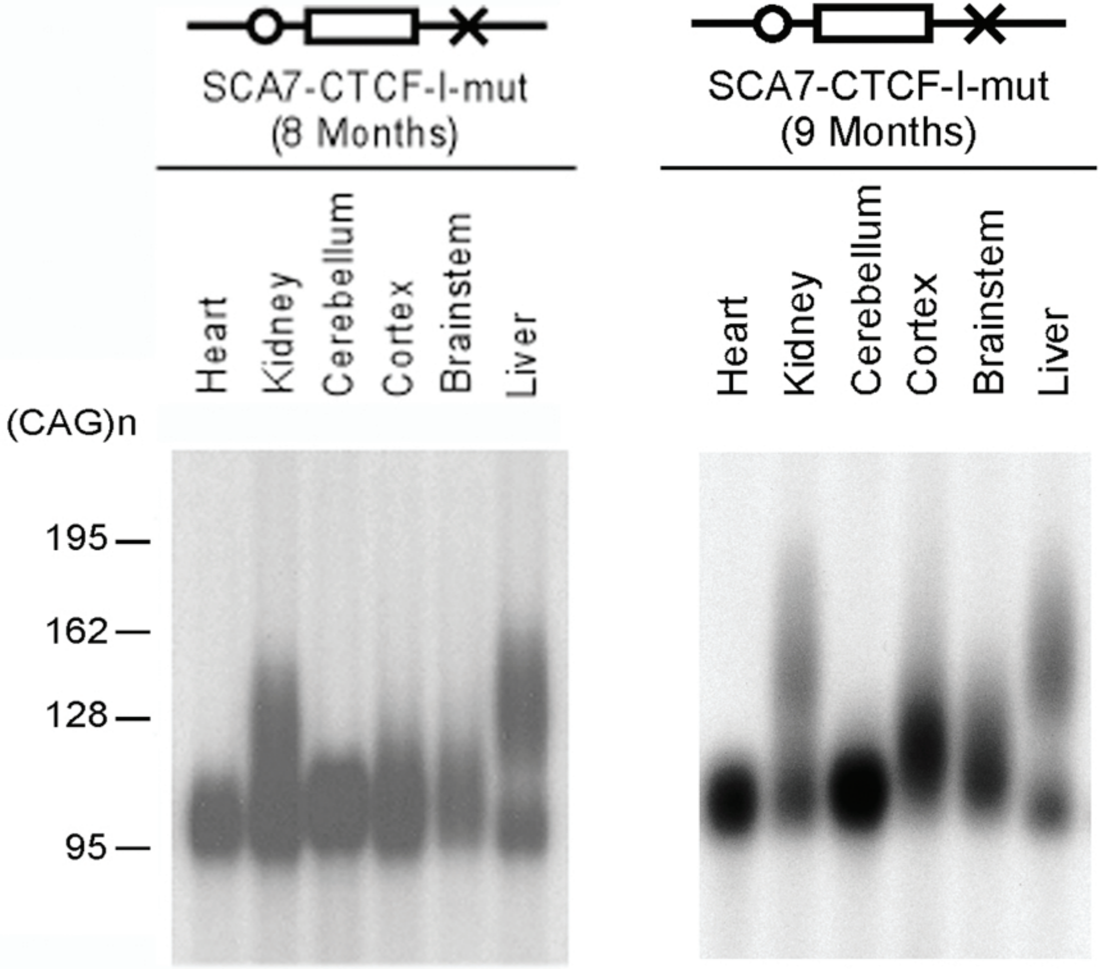

SCA7-CTCF-I-mut
(8 Months)
Heart
Kidney
Cerebellum
Cortex
Brainstem
Liver
SCA7-CTCF-I-mut
(9 Months)
Heart
Kidney
Cerebellum
Cortex
Brainstem
Liver
(CAG)n
195
162
128
95
