# Supplementary material for: CTCF cis-Regulates Trinucleotide Repeat Instability in an Epigenetic Manner: A Novel Basis for Mutational Hot Spot Determination
Source: PLoS Genet. 2008 Nov 14;4(11):e1000257. doi: 10.1371/journal.pgen.1000257 (PMC2573955; doi:10.1371/journal.pgen.1000257)
Supplement: Figure S6 — Amplicon for bisulfite sequencing for epigenotype determination. PCR amplification of bisulfite-converted genomic DNA for the fragment shown here was performed to derive CpG methylation status at the SCA7-CTCF-I binding site in murine tissues. Intron sequence is lowercase; exon sequence is uppercase. The SCA7-CTCF-I binding site is shown in blue. The thirteen CpG dyads included in the epigenotyping are shown, and the dyad with filled circles corresponds to a critical CTCF contact site, based upon footprinting analysis (see Figure 1C). (0.03 MB PDF) [file pgen.1000257.s006.pdf]

GTAGAGGCTT CCAAACTTCA TGGGAAGGAC Ggtgagtgtc **Exon 3**

ca**cg**ccctcc tccccccttc accccct**cgc g**accccctcc tctctcctcc
1 2 3

●●

cctcccccct **gcccccctcc tgtgaccc<u>g</u>c cccctcgagg ggcagagatg**
4 5

ctatcgtttg ctgggttgcg gaa**cgcg**gag gtgcccacac ctacccc**cg**tg **Intron 3**
6 7 8 9 10

cgtg**cg**tgag tgtg**cg**tcac actcctggcc actgacctgc ctc
11 12 13
